# An Exploratory Study of Civic Engagement and Participation Among a Panel of Adults Ages 85+

**DOI:** 10.1093/geroni/igaa057.2074

**Published:** 2020-12-16

**Authors:** John Rudnik, Taylor Patskanick, Julie Miller, Lisa D’Ambrosio, Joseph Coughlin

**Affiliations:** 1 MIT, Cambridge, Massachusetts, United States; 2 MIT AgeLab, Cambridge, Massachusetts, United States

## Abstract

The past twenty years have seen a surge in public attention devoted to increasing civic participation opportunities for older adults in the United States. At the same time, technology has transformed the way that information related to political and social issues is shared. A relatively small body of research has explored how older adults use technology-mediated platforms for political participation. In this study, the 85+ Lifestyle Leaders were surveyed to understand their experiences of civic engagement and participation. Responses to a questionnaire (N = 24) and focus groups (N = 22) indicate that participants are interested in and feel knowledgeable about social and political issues, but some forms of participation have decreased. Findings also suggest that there are opportunities for technology to facilitate engagement with and participation in social and political issues, for adults ages 85 and over. Issues of equity and accessibility will be highlighted in this presentation.

